# Comparative efficacy and safety of second- and later-line BRAF-targeted regimens in BRAF V600E–mutant metastatic colorectal cancer: A systematic review and network meta-analysis

**DOI:** 10.1097/MD.0000000000051468

**Published:** 2026-11-06

**Authors:** Muhammad Arsalan, F.N.U. Sawaira, Irtaza Hassan, Taimoor Hassan, Mavra Khan, Muhammad Yasir, Shariq Hayat, Muhammad Junaid, Hassaan Qazi, Sarim Hassan Shahab, Aizaz Anwar, Owais Gul, Mohammad Al Diab Al Azzawi

**Affiliations:** b Khyber Girls Medical and Dental College, Peshawar, Pakistan; c Bakhtawar Amin Medical and Dental College, Multan, Pakistan; d King Edwards Medical University, Lahore, Pakistan; e Bacha Khan Medical College, Mardan, Pakistan; f Khyber Medical College, Peshawar, Pakistan; a St Lukes Hospital, Chesterfield, Missouri; g Bannu Medical College, Bannu, Pakistan; h Nishtar Medical University, Multan, Pakistan; i Peshawar Medical College, Peshawar, Pakistan; j United Health Services Wilson Medical Center, New York; k Faculty of Medicine, The National Ribat University, Khartoum, Sudan.

**Keywords:** BRAF V600E, cetuximab, encorafenib, metastatic colorectal cancer, network meta-analysis, overall survival, progression-free survival

## Abstract

**Background:**

BRAF V600E mutations occur in approximately 8 to 12% of metastatic colorectal cancer (mCRC) cases and define an aggressive phenotype with poor prognosis. While the BEACON CRC trial established encorafenib-based combinations as effective second-line options, direct head-to-head comparisons among available BRAF-targeted regimens are lacking. This network meta-analysis (NMA) compared the relative efficacy and safety of second- and later-line BRAF-targeted regimens in BRAF V600E–mutant mCRC.

**Methods:**

PubMed, Embase, Cochrane CENTRAL, and ClinicalTrials.gov were searched from inception through January 2026. Eligible studies enrolled adult patients with BRAF V600E–mutant mCRC receiving second- or later-line BRAF-targeted therapy and reported time-to-event survival outcomes. A frequentist NMA was conducted under a random-effects model using the *netmeta* package in R. Hazard ratios (HRs) with 95% confidence intervals (CIs) were pooled for progression-free survival (PFS) and overall survival (OS). Where HRs were unavailable, individual patient data were reconstructed from Kaplan–Meier curves using the Guyot method. Treatment ranking used P-scores, with irinotecan plus cetuximab as the reference comparator.

**Results:**

Four trials enrolling 922 patients across 9 treatment arms were included. All 4 experimental regimens demonstrated significant PFS benefit over irinotecan plus cetuximab. Encorafenib plus cetuximab plus alpelisib ranked highest for PFS (HR 0.33, 95% CI 0.15–0.71; P-score 0.789), followed by encorafenib plus binimetinib plus cetuximab (EBC; HR 0.38, 95% CI 0.29–0.49), encorafenib plus cetuximab (EC; HR 0.40, 95% CI 0.31–0.50), and vemurafenib plus irinotecan plus cetuximab (HR 0.50, 95% CI 0.32–0.77). For OS, only EBC (HR 0.52, 95% CI 0.39–0.69) and EC (HR 0.59, 95% CI 0.46–0.76) achieved statistically significant benefits. No significant difference was detected between EBC and EC on either endpoint. No between-study heterogeneity was detected (I^2^ = 0%). In the exploratory safety network, encorafenib plus cetuximab was the only encorafenib-based regimen associated with significantly fewer grade ≥ 3 adverse events than irinotecan plus cetuximab (odds ratio 0.67, 95% CI 0.47–0.96).

**Conclusion:**

These findings position encorafenib-based therapy, and encorafenib plus cetuximab in particular, as the reference standard for previously treated BRAF V600E–mutant mCRC, while underscoring that the incremental value of more intensive regimens must be weighed against tolerability and confirmed in adequately powered trials.

## 1. Introduction

Colorectal cancer remains a leading cause of cancer-related morbidity and mortality worldwide, with a substantial proportion of patients presenting with or progressing to metastatic colorectal cancer. Among its molecular subtypes, the BRAF V600E mutation defines a distinct and clinically aggressive phenotype, characterized by poor prognosis, limited responsiveness to conventional chemotherapy, and shortened overall survival.^[1,2]^

Initial attempts to target this mutation using selective BRAF inhibitors yielded disappointing results in colorectal cancer, in contrast to other malignancies. Preclinical and clinical studies demonstrated that inhibition of BRAF leads to rapid reactivation of the MAPK pathway through compensatory signaling via EGFR, thereby attenuating therapeutic efficacy.^[3,4]^

Subsequent clinical strategies have focused on combination regimens targeting multiple nodes within this pathway. The pivotal BEACON CRC trial demonstrated that the combination of encorafenib and cetuximab, with or without binimetinib, significantly improved survival outcomes compared to standard chemotherapy in previously treated patients.^[5]^ Updated analyses from the same program further established the encorafenib–cetuximab doublet as a new standard of care, with comparable efficacy to the triplet combination.^[6]^ Both references^[5,6]^ derive from the BEACON CRC program and should be understood as analyses of the same trial rather than independent studies.

Despite these advances, uncertainty persists regarding the relative efficacy of the available BRAF-targeted regimens in the second- and later-line setting, particularly those incorporating additional pathway inhibitors such as PI3K inhibitors and MEK inhibitors. Direct head-to-head comparisons between these regimens are lacking, and the existing evidence base spans trials that differ substantially in design, including both randomized controlled trials and non-randomized phase Ib dose-escalation studies, as well as in patient populations and endpoints, thereby limiting cross-study interpretability.^[6,7]^

In this landscape, where progress is evident, yet the hierarchy of treatment efficacy remains indistinct, there exists a clear need for comparative synthesis. Therefore, this study performs a network meta-analysis of clinical trials evaluating second- and later-line BRAF-targeted regimens in BRAF V600E–mutant metastatic colorectal cancer. By integrating both direct and indirect evidence, this analysis aims to provide a robust comparative framework to inform clinical decision-making and optimize therapeutic sequencing.

## 2. Materials and methods

### 2.1. Protocol and reporting standards

This network meta-analysis was conducted in accordance with the Cochrane Handbook for Systematic Reviews of Interventions^[8]^ and has been reported in line with the Preferred Reporting Items for Systematic Reviews and Meta-Analyses (PRISMA) 2020 Guidelines^[9]^ to ensure transparency, accuracy, and reproducibility of the methodology and findings. The study protocol was registered in the International Prospective Register of Systematic Reviews (PROSPERO; registration number: CRD420261492649). Ethical approval and informed consent were not required because this study analyzed only aggregate data from previously published studies.

Through database search we identified 4 trials (phase I-III, randomized and non-randomized) that enrolled mCRC V600E patients in which first line therapy failed, and reported on doublet regimens combining a BRAF inhibitor with EGFR antibody and triplet, adding MEK inhibitor to doublet combination. We extracted Hazard ratios (HR) for PFS and OS, where it was explicitly stated, in other cases we used (Guyot et al)^[10]^ method. Frequentist Network Meta Analysis was performed on Random effect model to estimate pooled effects for each regimen relative to “Irinotecan + Cetuximab.” Additionally, this review was conducted in alignment with the AMSTAR 2 (A MeaSurement Tool to Assess systematic Reviews) checklist.^[11]^

### 2.2. Search strategy and data sources

A comprehensive literature search was performed in PubMed, Embase, Cochrane CENTRAL, and ClinicalTrials.gov from database inception to January 2026. The search strategy utilized a combination of Medical Subject Headings (MeSH) terms and keywords:

(“BRAF V600E” OR “BRAF mutation”) AND (“metastatic colorectal cancer” OR “mCRC”) AND (“BRAF inhibitor” OR “encorafenib” OR “vemurafenib” OR “dabrafenib” OR “targeted therapy”) AND (“second-line” OR “later-line” OR “previously treated”) AND (“randomized controlled trial” OR “RCT”).

### 2.3. Eligibility criteria

Studies were eligible if they: enrolled adults aged 18 years or older with histologically confirmed metastatic colorectal adenocarcinoma harboring a BRAF V600E mutation; evaluated a BRAF-targeted regimen, alone or in combination with an EGFR inhibitor, MEK inhibitor, PI3K inhibitor, or chemotherapy; included patients receiving treatment in the second-line or later-line setting after at least 1 previous systemic therapy; included a comparator treatment that allowed connection within the treatment network; and reported overall survival, progression-free survival, or sufficient information for extraction or reconstruction of time-to-event outcomes. Eligibility was structured according to the PICOS framework: the population comprised adults (≥18 years) with histologically confirmed BRAF V600E–mutant metastatic colorectal adenocarcinoma who had received at least 1 prior line of systemic therapy for metastatic disease; the intervention was any BRAF-targeted regimen (BRAF inhibitor alone or combined with an EGFR, MEK, or PI3K inhibitor, with or without chemotherapy); the comparator was any network-connectable regimen, most commonly irinotecan plus cetuximab or FOLFIRI plus cetuximab; the outcomes were overall survival and progression-free survival, with safety outcomes where reported; and eligible study designs were randomized controlled trials and prospective comparative studies. No language or geographic restrictions were applied.

Randomized controlled trials and prospective comparative clinical studies were eligible. We excluded first-line-only studies, single-arm studies without a network-connectable comparator, studies without separate BRAF V600E-mutant mCRC data, duplicate publications from the same trial population, reviews, editorials, case reports, protocols, preclinical studies, and studies without extractable efficacy or safety data. When multiple publications reported the same trial, the most complete and mature dataset was retained to avoid double counting.

### 2.4. Outcomes

The primary efficacy outcome was overall survival. Secondary efficacy outcomes included progression-free survival. Prespecified safety outcomes were grade 3 or higher treatment-emergent adverse events, serious adverse events, and adverse events leading to treatment discontinuation. Hazard ratios with 95% confidence intervals were extracted for survival outcomes. Odds ratios with 95% confidence intervals were calculated for dichotomous safety outcomes. Standard deviations were calculated using Meta-Analysis Accelerator.

### 2.5. Study selection and data extraction

Two reviewers independently screened titles and abstracts, assessed potentially eligible full-text articles, and selected studies according to the predefined eligibility criteria. Disagreements were resolved by discussion and, when necessary, adjudication by a third reviewer.

Two reviewers independently extracted data using a standardized form. Extracted information included first author, publication year, trial name and registration number, country, study phase and design, pathological type and molecular eligibility criteria (all trials enrolled BRAF V600E–mutant colorectal adenocarcinoma), treatment arms and doses, sample size, median age, sex distribution, prior treatment exposure, ECOG performance status, follow-up duration, and efficacy and safety outcomes. Efficacy outcomes included hazard ratios and 95% confidence intervals for progression-free survival and overall survival, or the data required to reconstruct them where these were not directly reported. Safety data sought included grade 3 or higher treatment-emergent adverse events, serious adverse events, and adverse events leading to treatment discontinuation; these were extracted where reported, and their availability across trials is detailed in the safety analysis.

For multiple publications from the same trial, only the most complete or most mature report was included for each outcome to prevent double counting of participants.

### 2.6. Risk of bias assessment

Two reviewers independently assessed the risk of bias of included studies. Randomized controlled trials were evaluated using the Cochrane Risk of Bias 2 tool across 5 domains: bias arising from the randomization process, deviations from intended interventions, missing outcome data, measurement of the outcome, and selection of the reported result. Non-randomized comparative studies were evaluated using the ROBINS-I tool across the domains of confounding, participant selection, classification of interventions, deviations from intended interventions, missing data, outcome measurement, and selection of the reported result. Disagreements were resolved by consensus or consultation with a third reviewer.

### 2.7. Statistical analysis

All statistical analyses were performed using the netmeta package^[12]^ (version 3.2.0) in R statistical software (R Foundation for Statistical Computing, Vienna, Austria), implementing the frequentist graph-theoretical approach to network meta-analysis as described by Rücker and colleagues.^[13]^ The network meta-analysis was conducted under a random-effects model, with between-study variance (τ^2^) estimated using the DerSimonian–Laird method,^[14]^ and I^2^ calculated based on Cochran’s Q statistic.^[15,16]^ The effect measure was the hazard ratio (HR) with its 95% confidence interval (95% CI), analyzed on the natural logarithmic scale; log-transformed HRs and their standard errors served as the inputs for all pooling procedures.^[17]^

Where hazard ratios and their 95% confidence intervals were explicitly reported in the source publications, these were extracted directly and log-transformed to log-HR and standard error of log-HR for input into the NMA model. For trials where individual patient data or directly reported HRs were not available, HRs were obtained by reconstructing Individual-patient level data from digitized Kaplan–Meier curves using the method described by Guyot and colleagues.^[10]^

The reference treatment for all network comparisons was irinotecan plus cetuximab, which served as the common comparator arm across the included trials.

Treatment ranking was performed using P-scores, the frequentist analogue of the Bayesian Surface Under the Cumulative Ranking curve (SUCRA), as described by Rücker and Schwarzer.^[18]^

A supplementary pairwise meta-analysis was conducted for comparisons with direct evidence from more than 1 trial. These pairwise estimates were used to validate the corresponding NMA estimates and assess the consistency of the network. Statistical significance was defined at a two-tailed α = 0.05 throughout.

Comparative safety was evaluated by network meta-analysis using the same frequentist random-effects framework applied to the efficacy outcomes, with irinotecan plus cetuximab as the reference. Grade ≥ 3 adverse events and adverse events leading to treatment discontinuation were analyzed as odds ratios; serious adverse events were not analyzed because overall counts with evaluable denominators were not consistently reported. Because adverse-event reporting was heterogeneous across trials, several comparisons were informed by single studies, and event counts were in some cases derived from reported percentages, the safety network estimates and treatment rankings are regarded as exploratory and are interpreted alongside the limitations.

## 3. Results

### 3.1. Search results

A systematic search of PubMed, Embase, Cochrane CENTRAL, and ClinicalTrials.gov from database inception through January 2026 yielded a total of 321 records (298 from databases and 23 from trial registries). Following automated deduplication and removal of records flagged as ineligible by automation tools, 299 records were carried forward for title and abstract screening. Of these, 143 records were excluded at the screening stage based on irrelevance to the PICO framework. A total of 156 reports were sought for full-text retrieval, of which 7 could not be obtained. The remaining 149 reports underwent full-text eligibility assessment. After detailed review, 144 reports were excluded for the following reasons: wrong study population (n = 56), failure to report eligible intervention or comparator (n = 39), and absence of time-to-event survival outcomes (n = 49). Ultimately, 4 studies, represented by 5 reports, met all eligibility criteria and were included in the final network meta-analysis. The complete study selection process is illustrated in the PRISMA flow diagram (Fig. 1).

**Figure 1.**
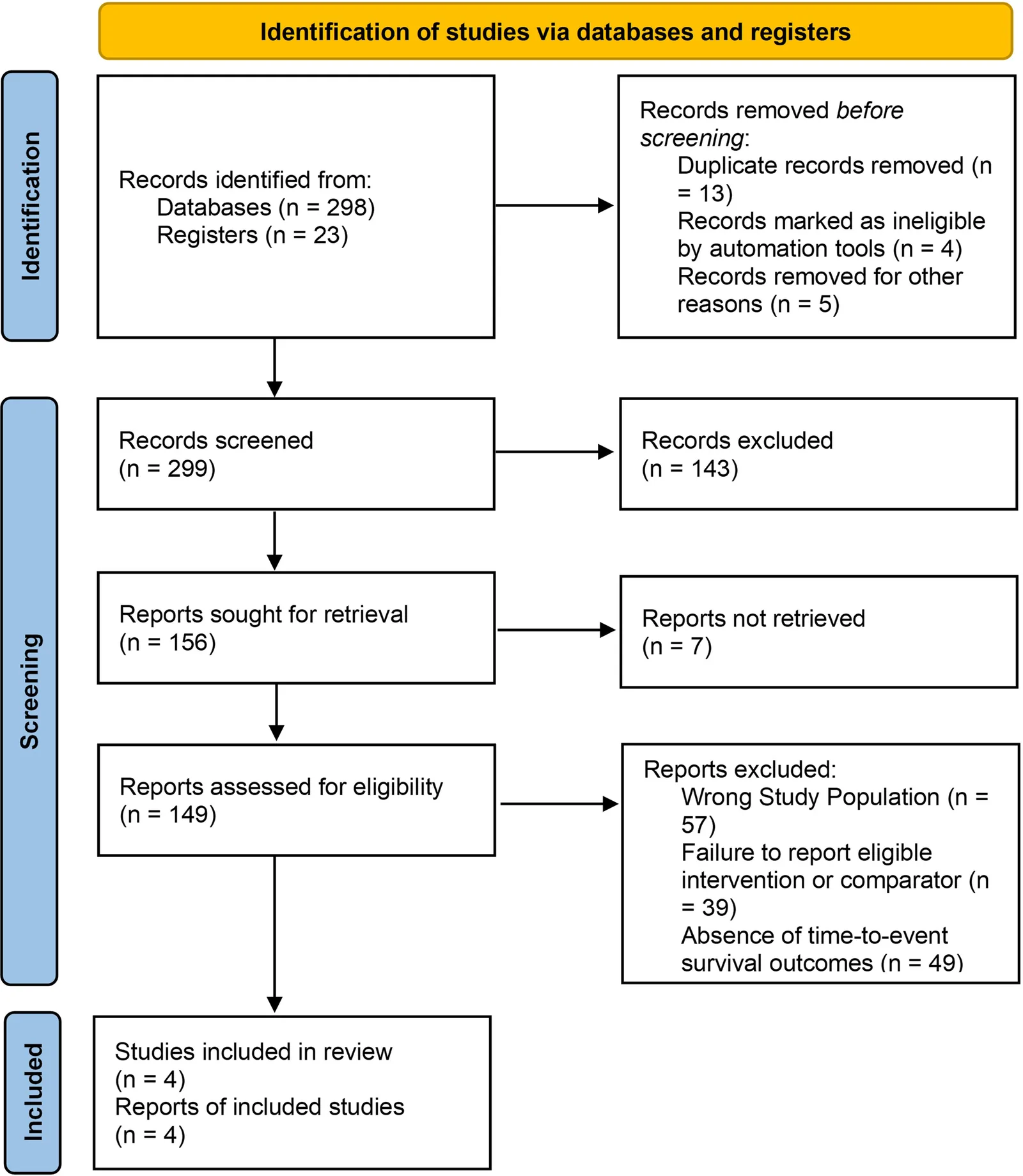
PRISMA 2020 flow diagram of study identification, screening, and selection for the network meta-analysis. PRISMA = preferred reporting items for systematic reviews and meta-analyses.

### 3.2. Quality assessment

Risk of bias was assessed for the 3 randomized controlled trials using the Cochrane Risk of Bias 2 (RoB 2) tool, and the results are presented in Figure 2A. The BEACON CRC trial (Kopetz et al)^[5]^ and its updated analysis (Tabernero et al)^[6]^ were judged to be at low risk of bias across all assessed domains, including the randomization process, deviations from intended interventions, missing outcome data, measurement of the outcome, and selection of the reported result. The SWOG S1406 trial (Kopetz et al)^[19]^ was judged to be at low overall risk of bias, with low risk in all domains except measurement of the outcome, for which some concerns were raised. The NAUTICAL trial (Wang et al.)^[20]^ was rated as low risk in the domains of outcome measurement and selection of the reported result; however, some concerns were raised regarding the randomization process and missing outcome data, and the domain of deviations from intended interventions was judged to be at high risk arising from the open-label design. Because 1 domain was judged to be at high risk, NAUTICAL was rated as having a high overall risk of bias. The non-randomized phase Ib study by Van Geel et al was assessed using the ROBINS-I tool and was judged to have serious risk of bias, primarily because of non-randomized cohort allocation and potential confounding. Detailed ROBINS-I domain-level judgments are provided in Figure 2B.

**Figure 2.**
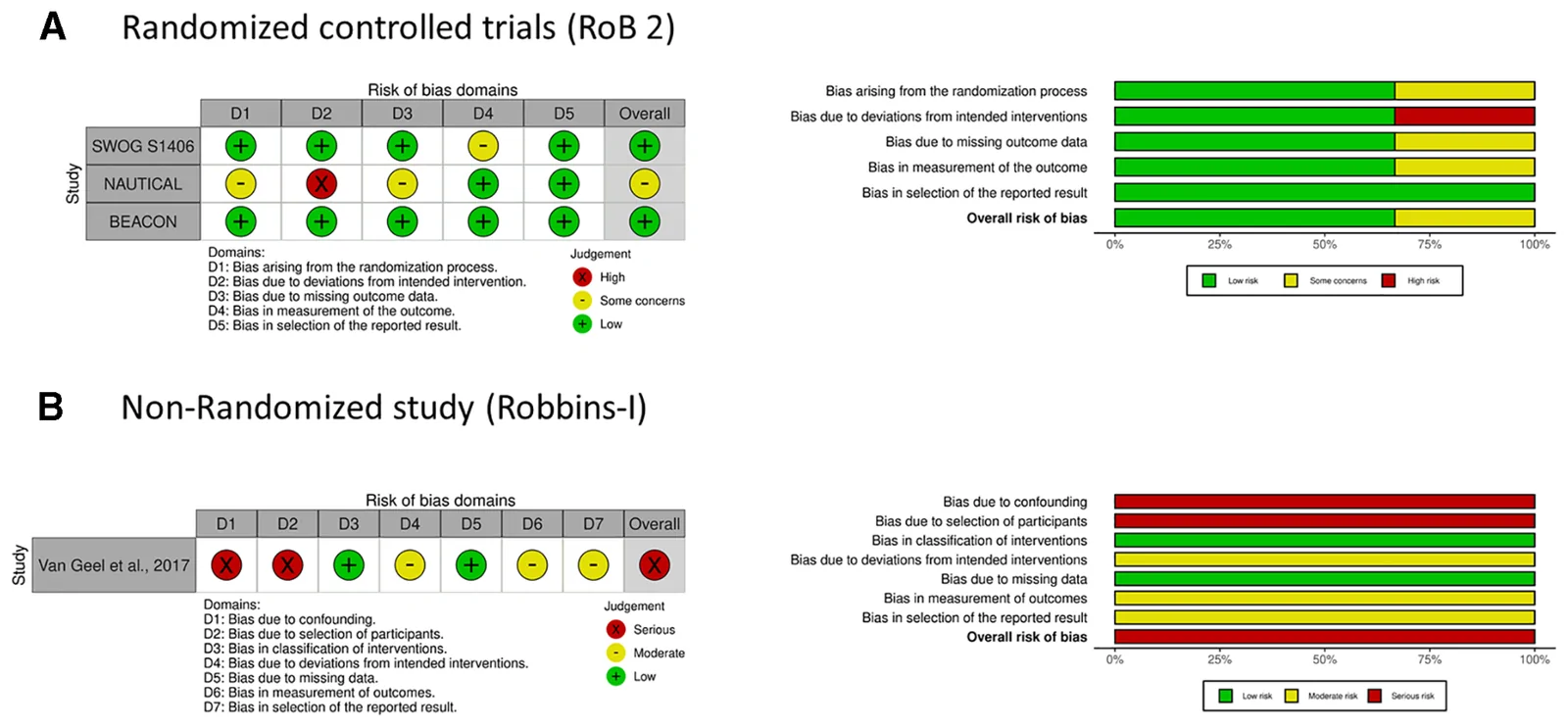
Risk-of-bias assessment of the included studies. (A) Cochrane Risk of Bias 2 traffic-light and summary plots for the randomized controlled trials, across domains D1 to D5: bias arising from the randomization process, bias due to deviations from intended interventions, bias due to missing outcome data, bias in measurement of the outcome, and bias in selection of the reported result. (B) ROBINS-I traffic-light and summary plots for the non-randomized study of van Geel et al., across domains D1 to D7: bias due to confounding, bias in selection of participants, bias in classification of interventions, bias due to deviations from intended interventions, bias due to missing data, bias in measurement of outcomes, and bias in selection of the reported result. RoB 2 = risk of bias 2 tool, ROBINS-I = risk of bias in non-randomized studies of interventions.

### 3.3. Study and Patient Characteristics

Four studies enrolling a total of 922 patients across 9 treatment arms were included in the final analysis. Table 1 presents the main characteristics of the included studies, and Supplementary Table 1, Supplemental Digital Content 1 provides detailed patient-level characteristics. The included studies span a range of trial designs: the BEACON CRC trial (Kopetz et al)^[5]^ was a phase III randomized open-label study; the SWOG S1406 trial (Kopetz et al)^[19]^ was a phase II randomized open-label study; the NAUTICAL trial (Wang et al)^[20]^ was a phase II randomized open-label study conducted in China, and Van Geel et al^[21]^ was a phase Ib open-label dose-escalation study. All trials were conducted in adult patients with confirmed BRAF V600E–mutant metastatic colorectal cancer who had received at least 1 prior line of systemic therapy. Treatment arms across the 4 studies included encorafenib plus cetuximab, encorafenib plus binimetinib plus cetuximab, encorafenib plus cetuximab plus alpelisib, irinotecan plus cetuximab (or FOLFIRI plus cetuximab), and vemurafenib plus irinotecan plus cetuximab.

**Table 1:** Main characteristics of the included studies.

Study	Trial name/NCT number	Country	Phase/design	Pathological/molecular eligibility	Treatment arms	Sample size	Median age, years	Female, %	Prior therapy
Kopetz et al^[5]^	BEACON CRC; NCT02928224	International/multicenter	Phase III, open-label, randomized, 3-arm trial	Adults with BRAF V600E-mutant metastatic colorectal cancer; ECOG performance status 0–1; progression after 1–2 prior regimens	Encorafenib + binimetinib + cetuximab; encorafenib + cetuximab; irinotecan or FOLFIRI + cetuximab	665 total; 224 triplet, 220 doublet, 221 control	61	49	One or two previous systemic regimens
Kopetz et al^[19]^	SWOG S1406; NCT02164916	United States	Phase II, open-label, randomized trial	Adults with BRAF V600E-mutant metastatic colorectal cancer; measurable disease; prior fluoropyrimidine and oxaliplatin exposure; no previous BRAF/EGFR inhibitor	Vemurafenib + irinotecan + cetuximab; irinotecan + cetuximab	106 randomized; 54 triplet, 52 control (50 evaluable per arm)	62	42	Previously treated metastatic disease; prior fluoropyrimidine and oxaliplatin required
Wang et al^[20]^	NAUTICAL; NCT05004350	China	Phase II, multicenter, open-label, randomized 2:1 trial	Adults with centrally confirmed BRAF V600E-mutant metastatic colorectal cancer; progression after 1–2 prior metastatic treatment lines; ECOG 0–1	Encorafenib + cetuximab; irinotecan + cetuximab or FOLFIRI + cetuximab	97 randomized; 65 doublet, 32 control	56	NR	One prior metastatic line: 77.3%; two prior lines: 22.7%
van Geel et al^[21]^	NCT01719380	International/multicenter	Phase Ib, open-label, non-randomized dose-escalation study	Adults with previously treated BRAF-mutant metastatic colorectal cancer	Encorafenib + cetuximab; encorafenib + cetuximab + alpelisib	54 total; 26 doublet, 28 alpelisib triplet	63 doublet; 59 alpelisib triplet	57.7 doublet; 64.3 alpelisib triplet	One prior line: 26.9% doublet, 35.7% triplet; ≥2 prior lines: 73.1% doublet, 64.3% triplet

BRAF = v-Raf murine sarcoma viral oncogene homolog B, CRC = colorectal cancer, ECOG = Eastern Cooperative Oncology Group, EGFR = epidermal growth factor receptor, FOLFIRI = folinic acid, fluorouracil and irinotecan, NCT = National Clinical Trial registry identifier, NR = not reported, SWOG = SWOG Cancer Research Network.

Patient-level characteristics were broadly comparable across trials, with a median age ranging from approximately 56 to 63 years. In the 3 trials reporting sex distribution, the proportion of female patients ranged from 42 to 64%. Primary tumor site was reported in 3 trials; colonic primary tumors were predominant in all arms of the BEACON CRC trial (100% colon), while rectal primaries constituted 6 to 12% of the SWOG S1406 population. ECOG performance status data were available for the van Geel et al, BEACON CRC, and SWOG S1406 trials; in all 3, the majority of patients had an ECOG PS of 0 or 1. Liver metastases were present in 56 to 66% of patients across reporting trials, and peritoneal involvement was documented in 19 to 32% of patients in the 2 trials that reported this variable. Most patients had received 1 or 2 prior lines of therapy, with the proportion having received exactly 1 prior line ranging from 27 to 77% across trials. These characteristics are detailed in Table S1, Supplemental Digital Content 1.

### 3.4. Progression free survival

A total of 4 trials (three RCTs and 1 non-randomized study), encompassing 5 treatment nodes, with Irinotecan + cetuximab serving as common comparator, formed a partially connected star-shaped network with 5 unique pairwise comparisons (Fig. 3). The network consisted of 4 different trial designs (d = 4), indicating design heterogeneity across trials. BEACON trial was only triple armed trial, which was incorporated as 3 pairwise comparisons (EBC vs IC, EC vs IC, and EBC vs EC) sharing a common control group. The correlation arising from the shared comparator was appropriately accounted for within the network meta-analysis model.

**Figure 3.**
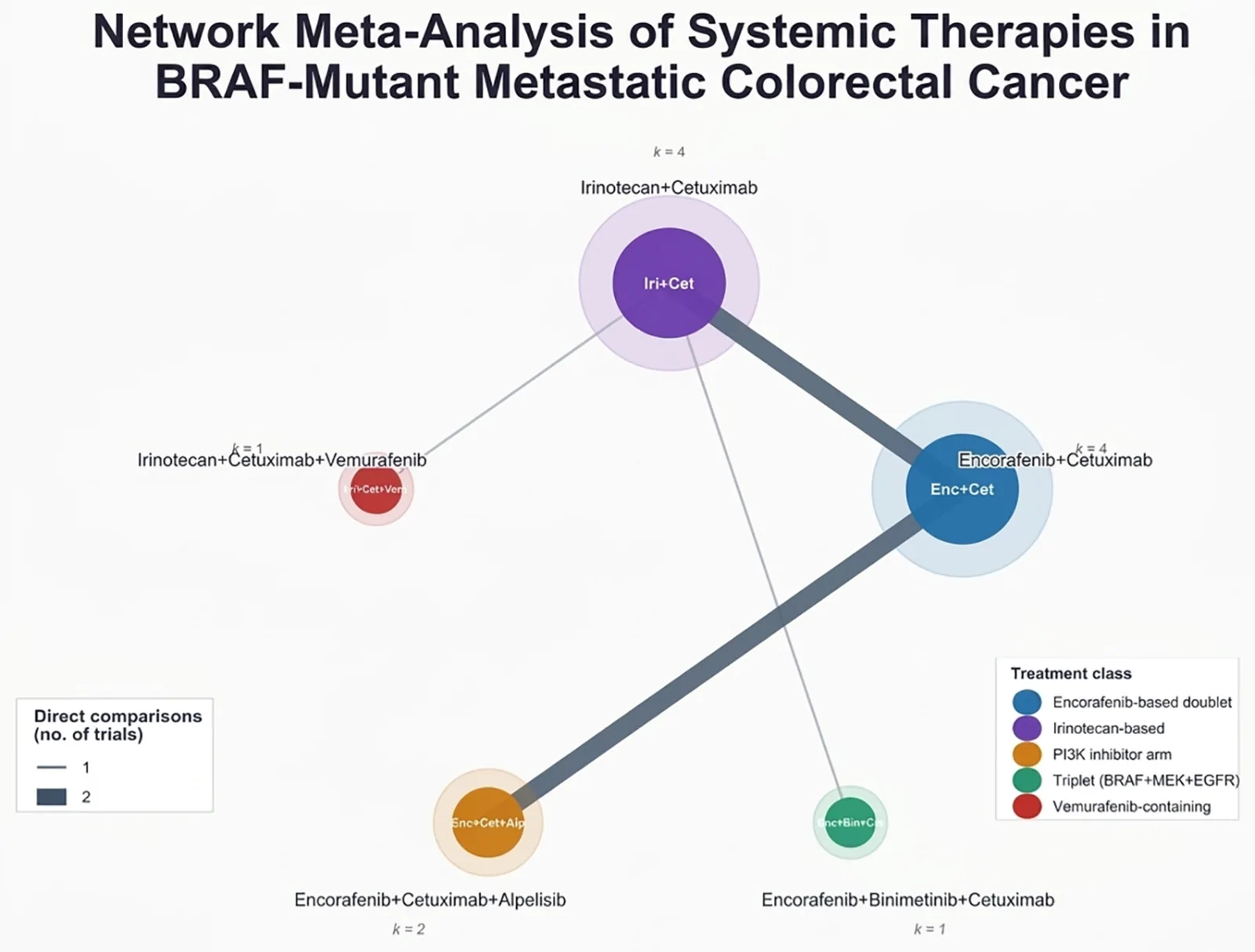
Network geometry of treatment comparisons in BRAF V600E-mutant metastatic colorectal cancer. Nodes represent treatment regimens and edges represent comparisons informed by direct evidence; edge thickness is proportional to the number of trials contributing to each comparison, and k denotes the number of trials contributing to each node.

Compared to Irinotecan + Cetuximab (IC), all evaluated regimens demonstrated statistically significant PFS benefit, with Encorafenib + cetuximab + Alpelisib (ECA) exhibited network derived HR 0.33 (95% CI 0.15–0.71) representing a 67% reduction in the risk of disease progression and Encorafenib + Binimetinib + Cetuximab (EBC) demonstrated HR 0.38 (95% CI 0.29–0.49) confirming a 62% reduction. Encorafenib + Cetuximab (EC) yielded pooled HR 0.40 (95% CI 0.31–0.50) estimated 60% reduction. Irinotecan + Cetuximab + Vemurafenib (VIC) with pooled HR 0.50 (95% CI 0.32–0.77) hinting at a 50% reduction in risk of progression or death (Fig. 4).

**Figure 4.**
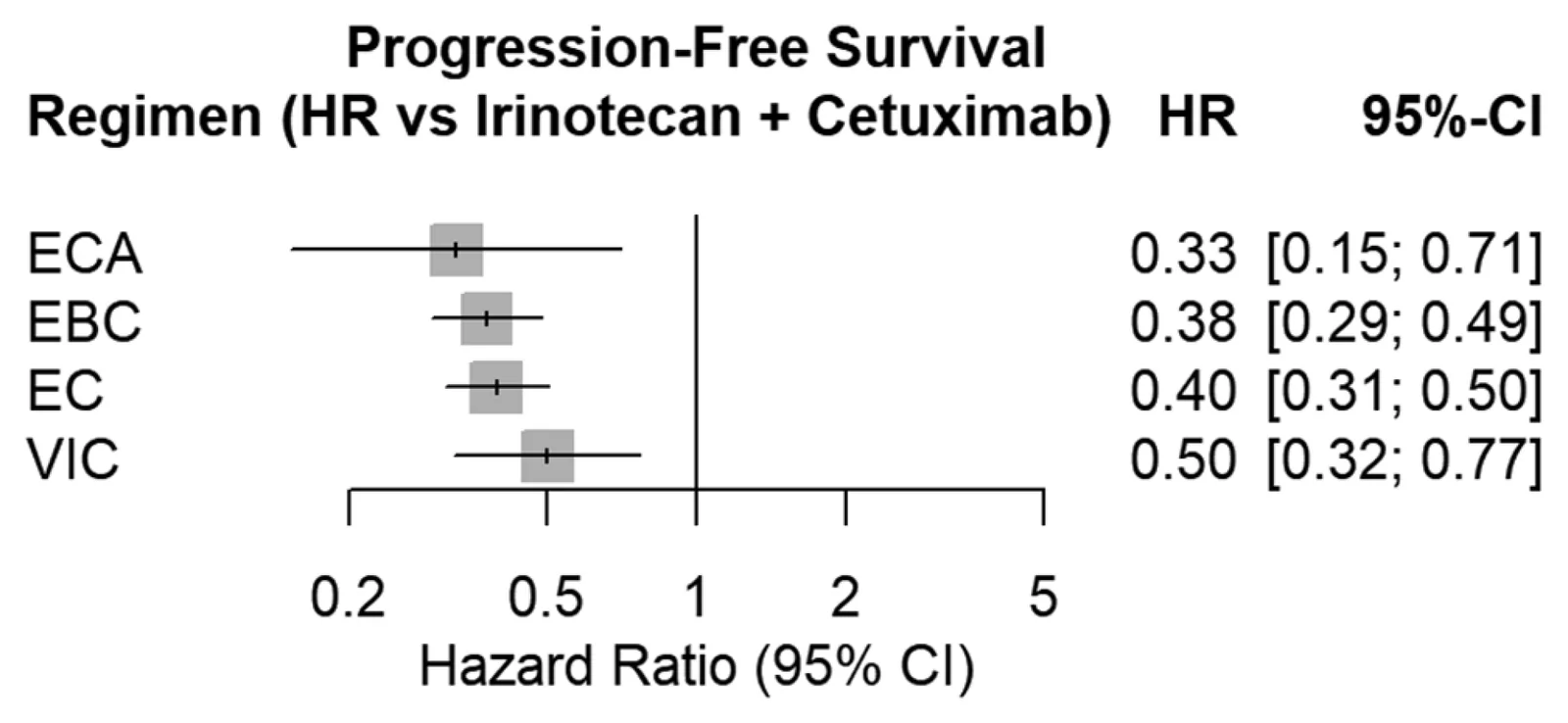
Forest plot of the network meta-analysis for progression-free survival, showing hazard ratios with 95% confidence intervals for each regimen relative to irinotecan plus cetuximab. Hazard ratios below 1 favor the experimental regimen. CI = confidence interval, EBC = encorafenib plus binimetinib plus cetuximab, EC = encorafenib plus cetuximab, ECA = encorafenib plus cetuximab plus alpelisib, HR = hazard ratio, IC = irinotecan plus cetuximab, VIC = vemurafenib plus irinotecan plus cetuximab.

Pairwise comparison from league table (Fig. 5) hinted the superiority of the Encorafenib-based regimen over the irinotecan-based therapy. *P*-score ranking analysis (Fig. 6) indicated that ECA had the highest probability of being the most effective regimen (p-score = 0.789).

**Figure 5.**
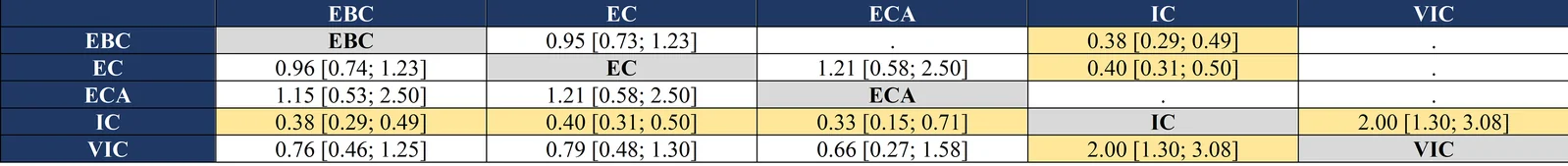
League table of pairwise comparisons for progression-free survival. Each cell reports the hazard ratio with its 95% confidence interval for the column-defining treatment relative to the row-defining treatment. CI = confidence interval, HR = hazard ratio; EBC = encorafenib plus binimetinib plus cetuximab, EC = encorafenib plus cetuximab, ECA = encorafenib plus cetuximab plus alpelisib, IC = irinotecan plus cetuximab, VIC = vemurafenib plus irinotecan plus cetuximab.

**Figure 6.**
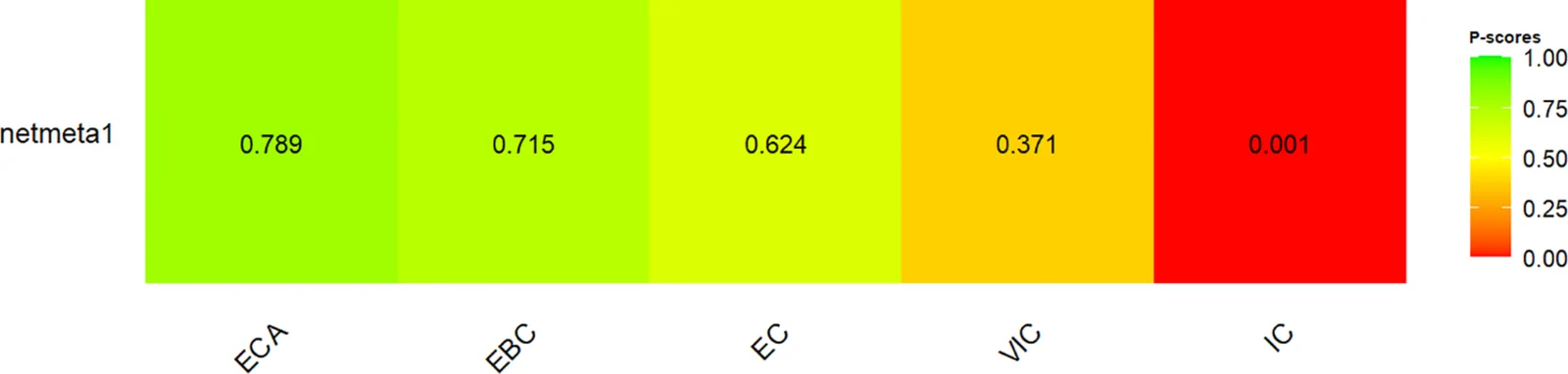
Treatment ranking for progression-free survival based on P-scores, the frequentist analogue of the surface under the cumulative ranking curve. Higher P-scores indicate a greater mean extent of certainty that a regimen is superior to the competing regimens. EBC = encorafenib plus binimetinib plus cetuximab, EC = encorafenib plus cetuximab, ECA = encorafenib plus cetuximab plus alpelisib, IC = irinotecan plus cetuximab, VIC = vemurafenib plus irinotecan plus cetuximab.

The network meta-analysis for PFS was conducted using frequentist random-effects model with DerSimonian-Laird estimator for between study variance (τ^2^). The model did not reveal any evidence of heterogeneity (I^2^ = 0%), indicating that treatment effects were consistent across included trials.

### 3.5. Overall survival

The network meta-analysis for Overall Survival (OS) was conducted with same network geometry (Fig. 3) as PFS, restricted to 3 RCTs with 4 regimens connected by IC. Analytical framework was also same, frequentist approach, random-effects model, DerSimonian–Laird estimator, using the three-trial network, in which the multi-arm BEACON trial was split into 3 pairwise comparisons sharing a common comparator.

Compared to Irinotecan + Cetuximab (IC), 2 regimens demonstrated statistically significant OS benefit, with Encorafenib + Binimetinib + cetuximab (EBC) exhibited network derived HR 0.52 (95% CI 0.39–0.69) representing a 48% reduction in the risk of death and Encorafenib + Cetuximab (EC) demonstrated HR 0.59 (95% CI 0.46–0.76) confirming a 41% reduction. The remaining regimen did not achieve a statistically significant OS benefit, with Irinotecan + Cetuximab + Vemurafenib (VIC) exhibiting HR 0.77 (95% CI 0.50–1.18). The encorafenib plus cetuximab plus alpelisib regimen could not be evaluated for overall survival because Van Geel et al did not report overall survival data (Fig. 7).

**Figure 7.**
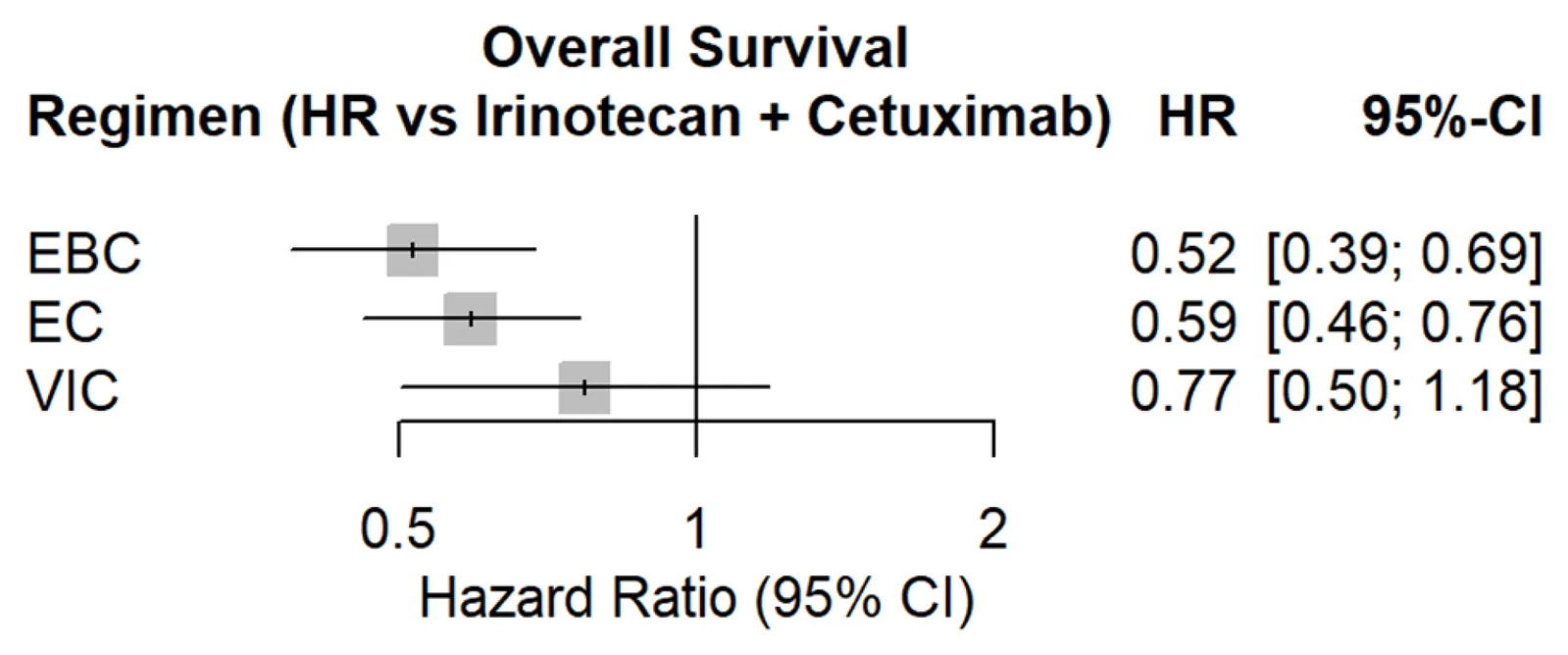
Forest plot of the network meta-analysis for overall survival, showing hazard ratios with 95% confidence intervals for each regimen relative to irinotecan plus cetuximab. Hazard ratios below 1 favor the experimental regimen. CI = confidence interval, EBC = encorafenib plus binimetinib plus cetuximab, EC = encorafenib plus cetuximab, ECA = encorafenib plus cetuximab plus alpelisib, HR = hazard ratio, IC = irinotecan plus cetuximab, VIC = vemurafenib plus irinotecan plus cetuximab.

Pairwise comparison from league table (Fig. 8) showed no statistically significant difference between the triplet (EBC) and the doublet (EC). *P*-score ranking analysis (Fig. 9) indicated that EBC had the highest probability of being the most effective regimen.

**Figure 8.**
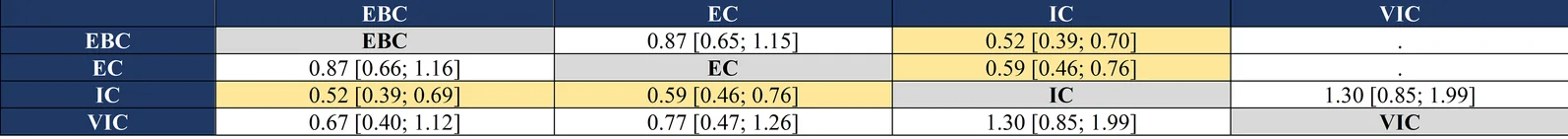
League table of pairwise comparisons for overall survival. Each cell reports the hazard ratio with its 95% confidence interval for the column-defining treatment relative to the row-defining treatment. CI = confidence interval, EBC = encorafenib plus binimetinib plus cetuximab, EC = encorafenib plus cetuximab, ECA = encorafenib plus cetuximab plus alpelisib, HR = hazard ratio, IC = irinotecan plus cetuximab, VIC = vemurafenib plus irinotecan plus cetuximab.

**Figure 9.**
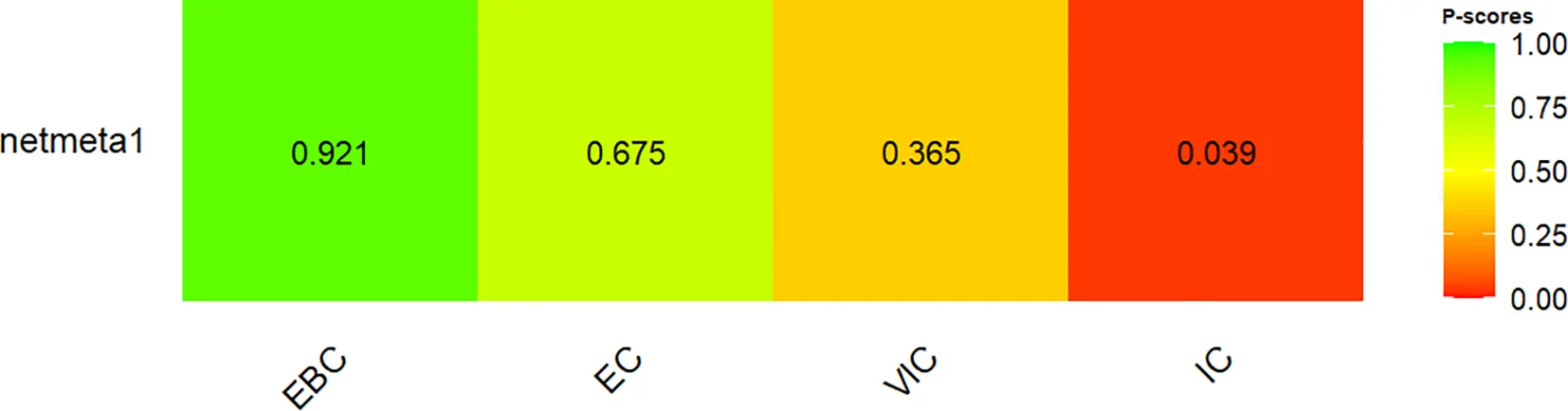
Treatment ranking for overall survival based on P-scores, the frequentist analogue of the surface under the cumulative ranking curve. Higher P-scores indicate a greater mean extent of certainty that a regimen is superior to the competing regimens. EBC = encorafenib plus binimetinib plus cetuximab, EC = encorafenib plus cetuximab, ECA = encorafenib plus cetuximab plus alpelisib, IC = irinotecan plus cetuximab, VIC = vemurafenib plus irinotecan plus cetuximab.

### 3.6. Safety outcomes

A safety network could be constructed for 2 outcomes. For grade ≥ 3 adverse events, all 4 trials contributed and the network was connected through the common comparator, with the encorafenib–cetuximab–alpelisib regimen linked only through the encorafenib–cetuximab node. Relative to irinotecan plus cetuximab, encorafenib plus cetuximab was associated with significantly fewer grade ≥ 3 events (OR 0.67, 95% CI 0.47–0.96), whereas encorafenib plus binimetinib plus cetuximab (OR 0.90, 95% CI 0.62–1.32) and encorafenib plus cetuximab plus alpelisib (OR 1.10, 95% CI 0.31–3.95) did not differ significantly from the comparator (Fig. 10A). Vemurafenib plus irinotecan plus cetuximab returned the lowest odds of grade ≥ 3 events (OR 0.22, 95% CI 0.08–0.60) and the highest P-score (Fig. 10B), but this estimate derives from a single trial (SWOG S1406) in which the control arm had an unusually high grade ≥ 3 rate (85%), and it should not be interpreted as evidence of superior tolerability. Between-study heterogeneity was estimated at zero (I^2^ = 0%), which reflects the limited number of comparisons rather than demonstrated consistency. For adverse events leading to discontinuation, 3 trials contributed; no regimen differed significantly from the comparator, and vemurafenib plus irinotecan plus cetuximab showed the highest odds of discontinuation (OR 3.24, 95% CI 0.96–11.00) (Fig. 11A and B). Full estimates and rankings are shown in safety league tables (Fig. 12).

**Figure 10.**
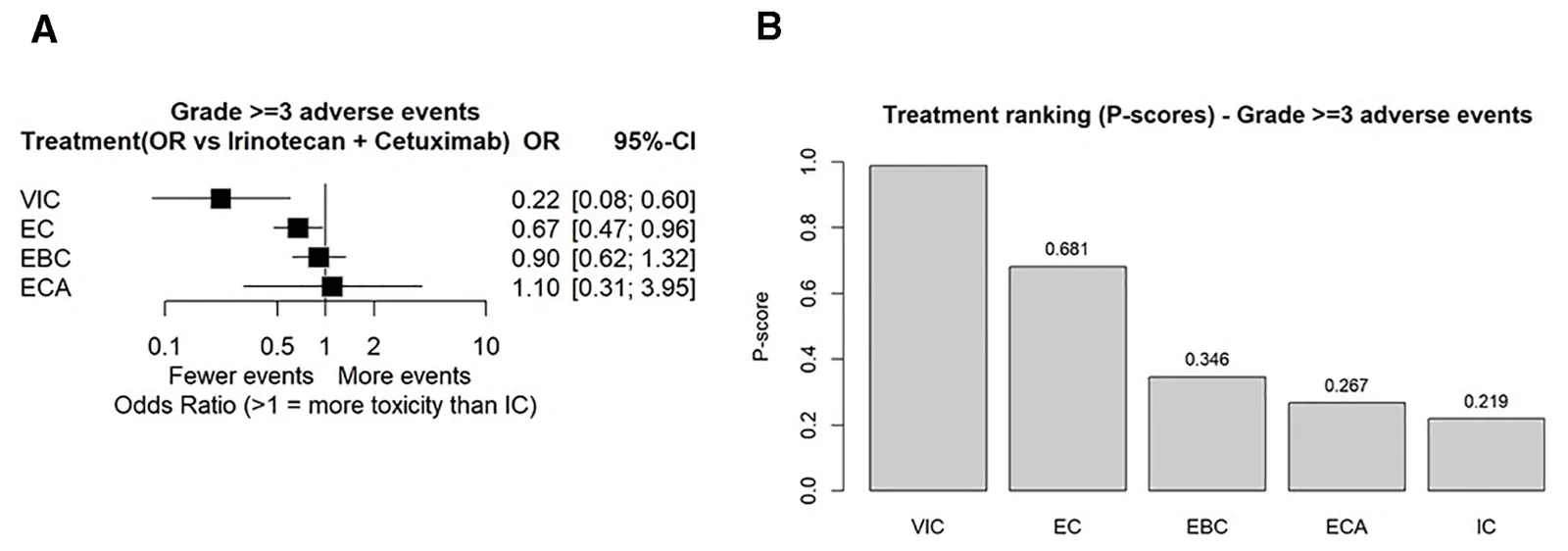
Comparative safety network meta-analysis for grade 3 or higher adverse events. (A) Forest plot of odds ratios with 95% confidence intervals for each regimen relative to irinotecan plus cetuximab; odds ratios above 1 indicate more events than the comparator. (B) Treatment ranking based on *P*-scores. CI = confidence interval, EBC = encorafenib plus binimetinib plus cetuximab, EC = encorafenib plus cetuximab, ECA = encorafenib plus cetuximab plus alpelisib, IC = irinotecan plus cetuximab, OR = odds ratio, VIC = vemurafenib plus irinotecan plus cetuximab.

**Figure 11.**
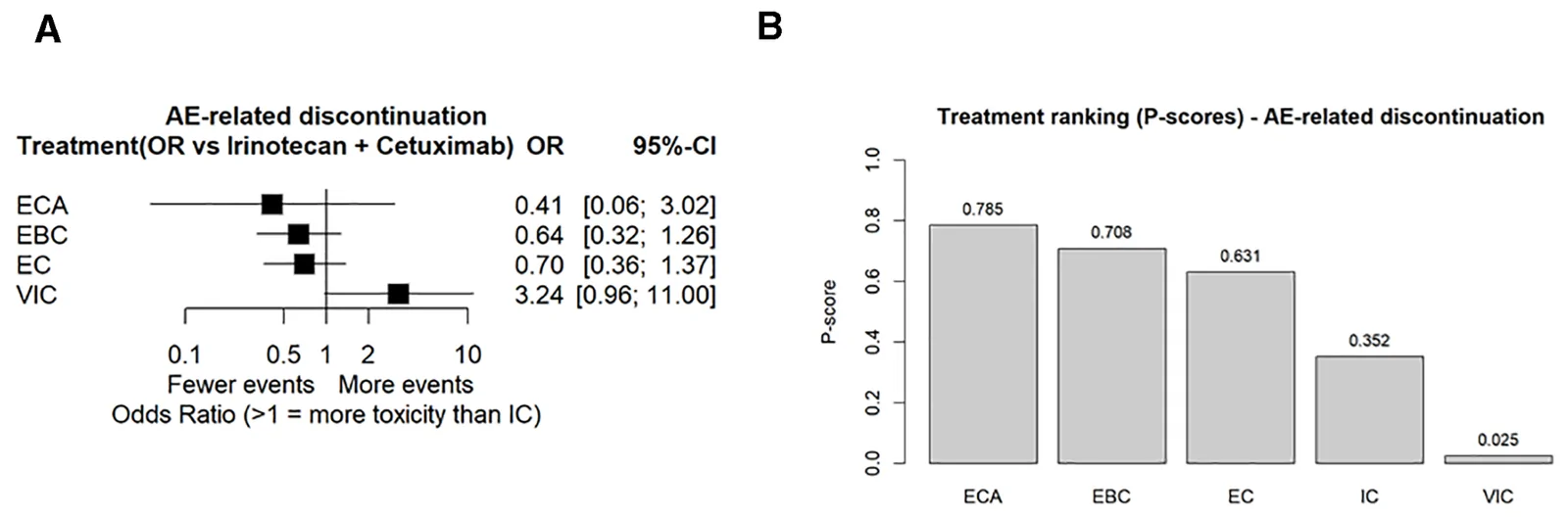
Comparative safety network meta-analysis for adverse events leading to treatment discontinuation. (A) Forest plot of odds ratios with 95% confidence intervals for each regimen relative to irinotecan plus cetuximab; odds ratios above 1 indicate more discontinuations than the comparator. (B) Treatment ranking based on P-scores. CI = confidence interval, OR = odds ratio, EBC = encorafenib plus binimetinib plus cetuximab, EC = encorafenib plus cetuximab, ECA = encorafenib plus cetuximab plus alpelisib, IC = irinotecan plus cetuximab, VIC = vemurafenib plus irinotecan plus cetuximab.

**Figure 12.**
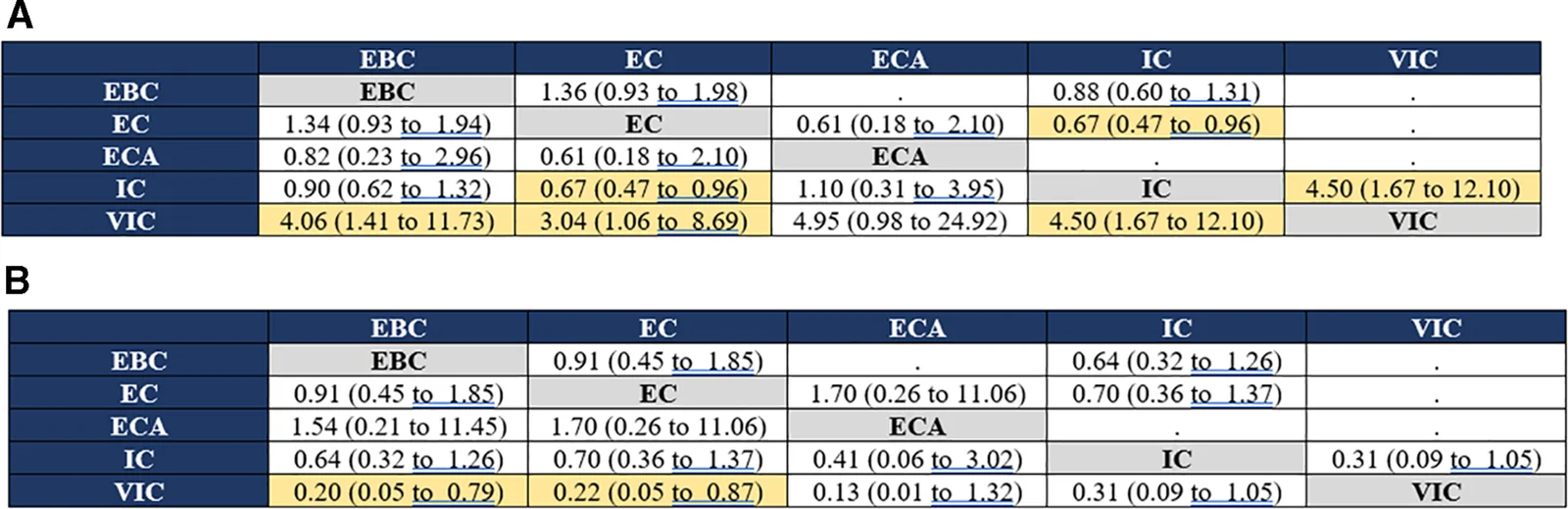
League tables of network meta-analysis results for the safety outcomes. (A) Grade 3 or higher adverse events. (B) Adverse events leading to treatment discontinuation. Each cell reports the odds ratio with its 95% confidence interval for the column-defining treatment relative to the row-defining treatment; shaded cells denote comparisons whose 95% confidence interval excludes 1, and a full stop denotes a comparison for which no estimate was obtainable. CI = confidence interval, EBC = encorafenib plus binimetinib plus cetuximab, EC = encorafenib plus cetuximab, ECA = encorafenib plus cetuximab plus alpelisib, IC = irinotecan plus cetuximab, OR = odds ratio, VIC = vemurafenib plus irinotecan plus cetuximab.

## 4. Discussion

This network meta-analysis synthesized evidence from randomized and non-randomized comparative clinical trials evaluating BRAF-targeted regimens in previously treated patients with BRAF V600E–mutant metastatic colorectal cancer.^[22,23]^ The inclusion of non-randomized evidence (Van Geel et al) represents a deliberate deviation from a strictly RCT-only eligibility framework, necessitated by the limited evidence base in this molecularly restricted population; this is acknowledged as a limitation of the current analysis. Using irinotecan plus cetuximab as the common comparator, our findings confirm that all 4 experimental regimens improved progression-free survival over the control. However, differences were noted when overall survival was evaluated, where a clinically relevant dissociation between PFS and OS rankings was observed with direct treatment implications for this patient group. Not all therapies that prolonged PFS did so for OS, emphasizing that treatment effectiveness in this disease must ultimately be measured by the harder endpoint of OS rather than disease control alone.^[19]^

The encorafenib plus cetuximab doublet and the encorafenib–binimetinib–cetuximab triplet emerged as the only regimens to deliver a statistically significant overall survival advantage, reducing the risk of death by approximately 41% and 48% respectively, consistent with what was reported in the primary and updated BEACON CRC analyses.^[5,6]^ Critically, no significant difference in either PFS or OS was detected between these 2 regimens in direct network comparison. While the triplet carried a numerically higher *P*-score for both endpoints, the overlapping confidence intervals and the absence of a statistically significant between-arm difference suggest that the doublet alone may offer a comparable survival benefit with a simpler regimen structure and a potentially more favorable tolerability profile.^[6,7]^ This is particularly relevant with respect to the emerging clinical experience with the BEACON CRC trial, where the doublet combination has performed similarly to the triplet combination in the primary analysis, which further supports the notion that the contribution of binimetinib addition to the encorafenib–cetuximab combination is limited.^[5]^

The encorafenib–cetuximab–alpelisib triplet presented the most intriguing and, in some ways, the most cautionary finding in this analysis. It ranked highest for PFS with a P-score of 0.79 and a hazard ratio of 0.33, but could not be evaluated for overall survival because Van Geel et al did not report overall survival data for this regimen. Critically, the effect estimates for the alpelisib triplet are derived entirely from indirect evidence, as this regimen has no direct head-to-head comparison against the control arm in any included trial; it connects to the network solely through the encorafenib doublet node. This structural position must be explicitly recognized when interpreting its rankings, as indirect estimates carry inherently greater uncertainty than those supported by direct evidence links. Its overall survival contribution therefore remains unknown and cannot be inferred from the present network. The biological plausibility of PI3K pathway inhibition augmenting disease control in BRAF V600E mCRC is supported by preclinical rationale demonstrating that PI3K/AKT pathway activation represents a key resistance mechanism to BRAF inhibitors,^[4,24]^ but the phase Ib and phase II data for the alpelisib triplet showed only modest incremental activity over the doublet.^[21]^ Whether alpelisib addition meaningfully alters the natural history of this aggressive subtype remains to be established in larger and more mature datasets. Until such evidence is available, the alpelisib triplet should be interpreted as a promising signal warranting further prospective evaluation rather than an established survival advantage ready for routine clinical adoption.

The vemurafenib combination performed least favorably among the experimental regimens. Although it achieved a statistically significant PFS benefit over the control, consistent with the SWOG S1406 findings,^[19]^ its overall survival hazard ratio of 0.77 (95% CI 0.50–1.18) did not indicate a statistically significant benefit. This estimate corresponds to the directly reported overall survival hazard ratio from SWOG S1406, which was used in the present analysis. Interpretation is nonetheless complicated by substantial crossover in that trial: approximately 42% of control arm patients crossed over to the experimental arm after progression, which would inflate control arm overall survival and attenuate any detectable benefit for vemurafenib. The crossover design of SWOG S1406 makes OS a particularly unreliable endpoint for this comparison, and the PFS signal, based on pre-crossover data, is therefore more informative for efficacy assessment in this specific trial. This is biologically plausible given that BRAF V600E mCRC exhibits rapid feedback reactivation of EGFR signaling upon BRAF inhibitor exposure,^[3,4]^ a mechanism that vemurafenib-based regimens may suppress less durably than the more pharmacodynamically potent encorafenib backbone. Regardless of the underlying explanation, this finding reinforces a critical principle in BRAF V600E mCRC: PFS improvement alone is an insufficient benchmark for treatment efficacy, and OS must remain the primary endpoint against which regimens are ultimately judged.^[19,25]^

The network consistency analysis provided important reassurance about the structural validity of these findings. The direct and indirect estimates of the encorafenib doublet and triplet against the control were in near-perfect mathematical agreement, which suggests that the transitivity assumption incorporated into the NMA was reasonably well met. However, it should be noted that a formal global inconsistency test, such as the design-by-treatment interaction test or Cochran’s Q for inconsistency, was not performed in this analysis, representing a methodological limitation. The agreement between direct and network estimates, while reassuring, serves as a proxy indicator of consistency rather than a formal statistical test of the inconsistency assumption. Future analyses with a larger evidence base should incorporate formal inconsistency testing. The selection of irinotecan plus cetuximab as the common comparator was not arbitrary but clinically deliberate. In the second- and later-line setting, BRAF V600E–mutant mCRC patients who progress on first-line chemotherapy have historically received irinotecan-based regimens combined with anti-EGFR antibodies, a combination that, despite modest efficacy, represents the most consistently used backbone across the randomized trials conducted in this population.^[5,6]^ By anchoring the network to this regimen, the NMA reflects the real-world treatment context in which these targeted therapies are deployed.

Comparative safety is essential when interpreting treatment rankings. Regimens incorporating additional MEK or PI3K pathway inhibition may offer improvements in disease control but can also increase treatment-related toxicity and the likelihood of treatment modification or discontinuation. Therefore, treatment selection should be individualized according to survival benefit, toxicity profile, performance status, comorbidities, previous therapy, and patient preferences. Because safety reporting was not fully uniform across the included trials, these findings should be interpreted cautiously.

In this analysis we extended the network framework to comparative safety, providing the first indirect comparison of grade ≥ 3 adverse events and treatment discontinuation across these regimens. The encorafenib–cetuximab doublet was the only encorafenib-based regimen associated with significantly fewer grade ≥ 3 adverse events than irinotecan plus cetuximab, while the binimetinib and alpelisib triplets did not differ significantly from the comparator. This pattern is consistent with the efficacy findings: the doublet combines a favorable tolerability signal with a proven survival benefit, reinforcing its position as a pragmatic standard in this setting, whereas the addition of a third pathway inhibitor did not yield a detectable tolerability advantage to offset its greater regimen complexity. These safety estimates, however, must be read with considerably more caution than the efficacy results, for reasons rooted in the structure of the evidence rather than the analysis itself. Adverse-event reporting was heterogeneous across trials in both granularity and definition; several comparisons were informed by a single study; some event counts were derived from reported percentages rather than explicit numerators; and serious adverse events could not be pooled at all because compatible overall counts were unavailable. The apparent absence of heterogeneity (I^2^ = 0%) should therefore not be mistaken for demonstrated consistency, as it chiefly reflects the small number of contributing comparisons. The most striking illustration of these limitations is the vemurafenib-containing regimen, which returned the lowest odds of grade ≥ 3 events yet the highest odds of treatment discontinuation – an internally contradictory profile. The favorable grade ≥ 3 estimate is an artifact of the single trial contributing that comparison (SWOG S1406), in which the control arm experienced an unusually high grade ≥ 3 event rate; it does not indicate that vemurafenib-based therapy is better tolerated, and the discontinuation signal is likely the more clinically faithful reflection of that regimen’s toxicity. Accordingly, these safety rankings should be regarded as exploratory and hypothesis-generating. A formal, adequately powered comparative safety evaluation, ideally derived from uniformly reported, patient-level adverse-event data, remains an important priority before firm tolerability distinctions can be drawn among these regimens.

## 5. Strengths and limitations

This analysis carries several methodological and clinical strengths. The network meta-analytic design allowed simultaneous comparison of multiple regimens in the absence of direct head-to-head trials, generating a clinically meaningful treatment hierarchy across this molecularly restricted population, in line with established methodological guidance for conducting and reporting NMAs.^[26]^ The assessment of OS and PFS as the prespecified primary and secondary endpoints, respectively was critical in establishing the PFS-to-OS dissociation as a key finding. Restricting the analysis to BRAF V600E–mutant mCRC as a molecularly defined subgroup, comprising approximately 8 to 12% of all mCRC cases and associated with distinctly aggressive clinical behavior,^[22,23]^ ensured clinical homogeneity and direct applicability to precision oncology practice.

These strengths notwithstanding, several limitations must be acknowledged. The network was constructed from 4 trials which, while representing the totality of available evidence in this population, limits the statistical power of indirect comparisons and increases the uncertainty of rank order estimates. *P*-scores in small networks are particularly sensitive to the inclusion or exclusion of individual studies and should be interpreted as probabilistic guidance rather than definitive rankings.^[27]^ The inclusion of a non-randomized phase Ib dose-escalation study (Van Geel et al) alongside randomized trials represents a deviation from a strictly RCT-based design and introduces potential selection bias, as cohort assignment in that study was sequential rather than randomized; cross-cohort comparisons should therefore be made with caution, as the authors themselves cautioned. The peripheral position of the alpelisib triplet in the network, connected to the common comparator solely through the encorafenib doublet, means its effect estimates are entirely indirect and carry greater imprecision than those of regimens with direct evidence links. Additionally, the NAUTICAL trial was conducted exclusively in a Chinese patient population, which may introduce a transitivity concern if there are meaningful biological or treatment-access differences in this subgroup relative to the predominantly Western populations enrolled in BEACON CRC and SWOG S1406. Although the low heterogeneity observed (I^2^ = 0%) is reassuring, this geographic restriction should be considered when generalizing the pooled doublet estimate. Patient-level heterogeneity across trials, including variation in prior lines of therapy, performance status, microsatellite instability status, and geographic differences in subsequent therapy access, may have introduced clinical heterogeneity that formal consistency tests cannot fully capture.^[28]^ Prediction intervals, which would convey the expected range of treatment effects in a new study setting and are recommended for random-effects NMA reporting, were not calculated in this analysis, representing a further reporting gap; given the small number of included trials, such intervals would likely be considerably wider than the confidence intervals presented, and their absence means the uncertainty of effects in future clinical contexts is underestimated. The maturity of OS data also varied across trials, and for regimens with shorter follow-up or fewer survival events, the OS estimates may not yet reflect true long-term outcomes. Finally, although we incorporated a comparative safety analysis in response to the recognized clinical importance of tolerability in this setting, the safety network was limited by heterogeneous and incompletely reported adverse-event data, several single-study comparisons, and the inability to pool serious adverse events; these safety estimates are therefore exploratory and were not powered to establish firm tolerability distinctions among regimens. This remains an important gap, given that MEK and PI3K inhibitor addition to the encorafenib–cetuximab backbone carries distinct and clinically meaningful toxicity profiles that must factor into individualized treatment decisions.^[6,7]^

## 6. Conclusion

In previously treated BRAF V600E–mutant metastatic colorectal cancer, encorafenib-based regimens demonstrated the most consistent survival benefit. Encorafenib plus cetuximab remains an important treatment option because it provides a survival benefit with a comparatively simpler treatment approach. Although intensified triplet regimens may improve progression-free survival, their overall survival benefit and safety profile require careful interpretation. Larger head-to-head trials are needed to define the optimal efficacy–safety balance among BRAF-targeted regimens.

## Author contributions

**Writing – original draft:** Muhammad Arsalan, FNU Sawaira, Irtaza Hassan, Taimoor Hassan, Mavra Khan, Muhammad Yasir, Shariq Hayat, Muhammad Junaid, Hassaan Qazi, Sarim Hassan Shahab, Aizaz Anwar, Owais Gul.

**Writing – review & editing:** Muhammad Arsalan, FNU Sawaira, Irtaza Hassan, Taimoor Hassan, Mavra Khan, Muhammad Yasir, Shariq Hayat, Muhammad Junaid, Hassaan Qazi, Sarim Hassan Shahab, Aizaz Anwar, Owais Gul, Mohammad Al Diab Al Azzawi.

**Conceptualization:** FNU Sawaira.

**Formal analysis:** Taimoor Hassan.

**Supervision:** Mohammad Al Diab Al Azzawi.


