# Supplementary material for: Comparative efficacy and safety of second- and later-line BRAF-targeted regimens in BRAF V600E–mutant metastatic colorectal cancer: A systematic review and network meta-analysis
Source: Medicine (Baltimore). 2026 Nov 6;105(45):e51468. doi: 10.1097/MD.0000000000051468 (PMC13747521; doi:10.1097/MD.0000000000051468)
Supplement: Supplementary file 1 [file medi-105-e51468-s001.pdf]

Supplemental Digital Content 1

Comparative Efficacy and Safety of Second- and Later-Line BRAF-Targeted Regimens in BRAF V600E–Mutant Metastatic Colorectal Cancer: A Systematic Review and Network Meta-analysis

Supplementary Table 1. Patient characteristics of included trials

Characteristic	van Geel 2017 EC (n = 26)	van Geel 2017 ECA (n = 28)	BEACON EBC (n = 224)	BEACON EC (n = 220)	BEACON IC (n = 221)	SWOG VIC (n = 50)	SWOG IC (n = 50)	NAUTICAL, all patients (n = 97)
Age, years, median (range)	63 (43–80)	59 (40–76)	62 (26–85)	61 (30–91)	60 (27–91)	61.9 (30–82)	59.7 (34–82)	56 (NR)
Female, n (%)	15 (58)	18 (64)	119 (53)	105 (48)	127 (57)	37 (74)	21 (42)	NR
**Primary tumour site**								
Right-sided colon, n (%)	NR	NR	126 (56)	110 (50)	119 (54)	30 (60)	36 (72)	NR
Left-sided colon, n (%)	NR	NR	79 (35)[a]	83 (38)[a]	68 (31)[a]	17 (34)	8 (16)	NR
Rectum, n (%)	2 (8)	3 (11)	—[a]	—[a]	—[a]	3 (6)	6 (12)	NR
Colon, side not specified, n (%)	24 (92)	25 (89)	—	—	—	—	—	NR
Both sides or unknown, n (%)	—	—	19 (8)	27 (12)	34 (15)	—	—	NR
**Performance status**								
ECOG / Zubrod PS 0, n (%)	8 (31)	18 (64)	116 (52)	112 (51)	108 (49)	23 (46)	24 (48)	NR
ECOG / Zubrod PS 1, n (%)	16 (62)	10 (36)	108 (48)	104 (47)	113 (51)	27 (54)	26 (52)	NR
ECOG / Zubrod PS 2, n (%)	2 (8)	0	0	4 (2)	0	0[d]	0[d]	NR
**Disease extent**								
Liver metastases, n (%)	15 (58)	16 (57)	144 (64)	134 (61)	128 (58)	28 (56)	33 (66)	55 (56.7)[b]
Peritoneal metastases, n (%)	5 (19)	8 (29)	—	—	—	16 (32)	16 (32)	—
≥3 organs involved, n (%)	—	—	110 (49)	103 (47)	98 (44)	—	—	47 (48.5)[b]
**Prior systemic therapy for metastatic disease**								
Prior adjuvant therapy only, n (%)	—	—	—	—	—	8 (16)	3 (6)	—
1 prior regimen, n (%)	7 (27)	10 (36)	146 (65)	146 (66)	145 (66)	25 (50)	27 (54)	75 (77.3)[b]
2 prior regimens, n (%)	8 (31)	14 (50)	78 (35)[c]	74 (34)	76 (34)[c]	17 (34)	20 (40)	22 (22.7)[b]
3 prior regimens, n (%)	5 (20)	1 (4)	—	—	—	—	—	—
>3 prior regimens, n (%)	6 (23)	3 (11)	—	—	—	—	—	—

Abbreviations: EBC = encorafenib + binimetinib + cetuximab; EC = encorafenib + cetuximab; ECA = encorafenib + cetuximab + alpelisib; ECOG = Eastern Cooperative Oncology Group; IC = irinotecan + cetuximab; NR = not reported; PS = performance status; SWOG = SWOG Cancer Research Network; VIC = vemurafenib + irinotecan + cetuximab. Dashes (—) indicate data not reported for that arm. [a]BEACON CRC did not report rectal primaries separately; rectal tumours are included within the left-sided colon category. [b]Reported for the overall NAUTICAL population (both arms combined); arm-level data were not available. [c]In BEACON CRC, this category includes one patient in the triplet (EBC) arm and one patient in the control (IC) arm who received more than two previous lines of therapy. [d]SWOG S1406 restricted eligibility to Zubrod performance status 0–1.
